# Effectiveness of Behavior Change Communications for Reducing Transmission Risks Among People Living with HIV in 6 Countries in Central America

**DOI:** 10.1007/s10461-014-0910-0

**Published:** 2014-10-05

**Authors:** Lung Vu, Benjamin Nieto-Andrade, Allison DiVincenzo, Jorge Rivas, Rebecca Firestone, Jennifer Wheeler, Sussy Lungo

**Affiliations:** 1 Population Services International, 1120 19th Street, NW, STE 600, Washington, DC 20036 USA; 2 Population Council, 4301 Connecticut Ave, NW, STE 280, Washington, DC 20008 USA; 3 Population Services International, Avenida 21 de Janeiro, Rua da Ex-Monua, Bairro Morro Bento II Luanda, Angola; 4 Pan American Social Marketing Organization (PASMO), 13 Calle 3-40, Zona 10, Edificio Atlantis, Nivel 13, Oficina 1305, Guatemala City, Guatemala

**Keywords:** People living with HIV, Central America, HIV risks, ART, Combination prevention, Effectiveness, Behavior change communications (BCC)

## Abstract

This first region-wide study (N = 2,818) aims to estimate prevalence of HIV-related risks (sexual behavior, HIV disclosure, number of sex partners, violence) and factors associated with these risks as well as evaluate a behavior change communications program targeted to PLHIV in 6 countries in Central America. After 2 years, the program achieved moderate coverage, with 21 % of the sample reporting exposure to interpersonal communications (IPC) and 52 % to mass media program components. The odds of condom use, HIV disclosure, and participation in a self-help group increased by 1.4–1.8 times with exposure to mass media. Exposure to IPC increased odds of condom use by 2.7 and participation in self-help groups by 4.4 times. In addition, being in HIV care or taking ART was associated with condom use and HIV-status disclosure. About 30 % experienced physical or sexual violence, and those who did were 4 times less likely to use condoms. Findings suggest that behavioral interventions for PLHIV can reduce HIV-transmission risks and increase access to care.

**Electronic supplementary material:**

The online version of this article (doi:10.1007/s10461-014-0910-0) contains supplementary material, which is available to authorized users.

## Resumen

Este primer estudio a nivel regional estima la prevalencia de riesgos de transmisión (comportamiento sexual, compartir el status de VIH, número de parejas sexuales, violencia) y los factores asociados a estos riesgos y también evalúa un programa de comunicación para el cambio de comportamiento dirigido a personas con VIH en Centroamérica. Después de 2 años, el programa logró cobertura moderada, donde 21 % reportó exposición a actividades de comunicación interpersonal (CIP) y 52 % al componente de medios masivos del programa. La probabilidad de uso del condón, de compartir el estatus de VIH con alguien más, y de participar en grupos de auto-apoyo se incrementó entre 1.4-1.8 veces con la exposición a medios masivos. La exposición a CIP incrementó la probabilidad de uso del condón 2.7 veces y de participar en grupos de auto-apoyo 4.4 veces. Asimismo, recibir atención sobre VIH o tomar ARV se asoció con uso del condón y con compartir el estatus de VIH con otras personas. Cerca del 30 % experimentó violencia física o sexual, y aquellos que la experimentaron reportaron una probabilidad 4 veces menor de usar condones. Los resultados sugieren que las intervenciones de cambio de comportamiento dirigidas a personas con VIH pueden reducir el riesgo de transmisión del VIH y aumentar el acceso a la atención.

## Introduction

Central America has a concentrated HIV epidemic with an estimated 148,500 people living with HIV (PLHIV) and 8,600 new infections (range between 4,600 and 25,200) annually [1]. The overall HIV prevalence in Central America varies with the highest prevalence in Belize (1.4 %), followed by Guatemala and Panama (0.7 %), El Salvador (0.6 %), Honduras (0.5 %), and Costa Rica and Nicaragua (0.3 %) [1]. Key populations at higher risk of HIV infection, including men who have sex with men (MSM), transgender women (TW), and female sex workers (FSWs), suffer a much higher HIV burden. HIV prevalence among MSM is estimated to range from 6.6 % in Nicaragua to 13.3 % in Guatemala [1] and among FSWs, from 2.2 % in Nicaragua to 9.7 % in Honduras [2].

Among PLHIV in Central America, recent studies show high levels of HIV-related risks such as modest levels of condom use [3–6]; having high prevalence of STIs, especially herpes simplex virus type 2 and syphilis [3–5]; and having multiple sex partners [4, 5], potentially accelerating HIV transmission. Many PLHIV do not seek care and treatment services or do not adhere properly to treatment [6], negating the impact of ART on viral load suppression, and thus diminishing the potential impact of treatment as prevention (TasP) [7]. In addition, PLHIV in Central American experience high levels of stigma and discrimination [4, 5], as well as violence and abuse [6]. A review of the literature on PLHIV worldwide reveals additional factors that impact risk behaviors, including low levels of HIV status disclosure [8], little social and psychological support [9], poor mental health, and low ART adherence [10].

The Positive Health Dignity Prevention (PHDP) framework, jointly developed by the Joint United Nations Program on HIV/AIDS (UNAIDS) and the Global Network of People Living with HIV (GNP+), promotes a comprehensive response to the needs of PLHIV. This framework helps PLHIV lead healthy lives and reduce the risk of transmitting HIV to others in a context which recognizes structural barriers to healthy behaviors and seeks to reduce stigma and discrimination [11]. PLHIV in the context of a concentrated epidemic, many of whom are sex workers, homosexual men, people who use drugs, or prisoners, are subjected to multiple levels of stigma and discrimination due to their HIV-positive status, their sexual identity, or their profession [12]. Many PLHIV are denied medical care, housing, and jobs due to their HIV-positive status [9, 13, 14]. Stigma and discrimination are found to affect physical and mental health and decrease HIV status disclosure and ART adherence [10, 12, 15, 16]. Further, external stigma and discrimination can be internalized into an individual’s self-concept, creating psychological distress and preventing PLHIV from accessing health care services and seeking social support [17].

To respond to the global effort in preventing HIV transmission and improving the health and well-being of PLHIV, the Pan American Social Marketing Organization (PASMO)—a member of Population Services International (PSI) network—has been implementing an intervention program among key populations, including MSM, Transgender Women (TW), FSWs, men at risk, and PLHIV since 2010 under the five year USAID-funded Combination Prevention Program. The USAID’s combination prevention approach is aligned with the PHDP framework described above, aiming to respond to the comprehensive needs of individuals living with HIV. Under this program, PASMO defined an essential package of interventions per target population under each of three combination prevention components: behavioral, biomedical, and structural. For PLHIV, the minimum package includes: (a) participation in at least three behavior change communications (BCC) interventions, including peer-led interpersonal communication (IPC) or online outreach with topics such as safer sex practices, condom access, adherence to ART, and nutrition; (b) referrals to screening and treatment of STIs, treatment for opportunistic infections, and access to ART programs; and (c) referrals to structural and complementary services such as family planning, support groups, legal support, and treatment for alcohol and drug use. Due to some limitations in the measurement of the two latter components (b and c), the evaluation (Objective 2 below) focuses on the BCC component (a).

There is limited data on PLHIV in Central America and a particular lack of understanding of intervention effectiveness to reduce HIV transmission and improve the well-being of PLHIV in Central America and worldwide. This large, region-wide quantitative study aims to: (1) Describe key socio-economic and behavioral characteristics of PLHIV in Central America, including condom use, HIV-status disclosure, access to care, and experiences with violence that are critical to the success of HIV prevention among PLHIV; and (2) Evaluate the midterm (2 years after the intervention) effectiveness of the BCC component of the Combination Prevention Program, assessing whether participating in PASMO’s prevention activities reduces risk behavior and increases access to services. HIV transmission in Central America can be mitigated with a greater understanding of the risk-related factors, intervention needs, and intervention effectiveness.

## Methods

### Study Design

A cross-sectional study was used, aiming to measure the differences in key behavioral outcomes (e.g. proportion of condom use, HIV status disclosure, multiple sexual partnerships, and participation in support groups) between exposed and non-exposed participants at the regional level. We used the most conservative sample size estimate, assuming 45 % in reporting of key indicators, 15 % difference in key indicators between exposed and unexposed groups, 95 % confidence interval and a power of 80 %. Other factors considered in the sample size estimate were the assumptions of 10 % nonresponse, and 15 % being exposed to the program. The final sample size was 2,838. The details of the sample size formula and justifications can be found elsewhere [18, 19].

### Recruitment Procedure

PLHIV aged 18 and older who resided in the study catchment areas at the time of the survey were eligible to participate in the study. Due to the sensitivity of the topic, we did not have the access to the patient records and thus no sampling frame was defined. Consequently, a convenience sampling strategy was used. First, we identified all of the clinics and organizations working with PLHIV in the program area. Second, key personnel at these locations were sensitized about the study and permission to conduct the survey was sought and granted. Doctors, nurses, and consented peer educators informed HIV-positive patients and clients about the purpose of the study and invited them to participate. If an individual was interested, he or she was then referred to a trained interviewer for administration of informed consent and a 30 min survey interview. We were able to reach 2818 PLHIV from September to December 2012. Details of the sample size for each country are described in the Supplemental Table 1.

### Measurements

The survey instrument was adapted from the AIDS Indicator Survey and the Global Network of People Living with HIV Survey (GNP+). The survey was conducted face-to-face and included questions on socio-demographic characteristics, HIV-related risks (i.e. condom use, HIV-status disclosure, and number of sex partners), participation in a support group, access to CD4 testing, access to ART treatment and exposure to PASMO’s behavior change communication activities (mass media and IPC).

Key outcomes for this paper were determined by programmatic objectives, including increased condom use at last sex, HIV status disclosure to sex partner, ART adherence, and current participation in a support group. HIV disclosure was measured by asking if the person disclosed his or her HIV status to the last sex partner during the last sexual encounter. Receiving ART was defined as currently taking daily ART as prescribed by an HIV physician. Adherence to ART was determined if the person was able to take the ART pills as instructed by his or her doctor without any missing dose within the last 30 days. This variable was restricted to the sub-sample of people who are on ART (N = 2,111).

A series of covariates hypothesized to influence these outcomes were included. Socioeconomic status (SES) was measured using a validated index developed by the Asociacion Mexicana de Agencias de Inteligencia (AMAI) [20]. The index uses household assets and educational levels of the primary breadwinner. Violence was measured by asking 4 questions about being physically, sexually, verbally, and psychologically abused within the past 12 months. Physical and sexual violence were collapsed into one variable for multivariate analysis. The same was applied to verbal and psychological abuse.

Exposure to PASMO’s behavior change communications intervention was measured in 3 variables: (1) exposure to 3 TV advertisements and (2) exposure to at least one IPC session via face-to-face chat with an outreach worker or peer educator in the past 12 months. Exposure to the third variable, Internet chat, was low and thus was not included in the analysis.

### Data Analysis

#### Factors Associated with Condom Use at Last Sex and HIV-Status Disclosure

Univariate analysis was used to describe the population characteristics, HIV-related risks, and levels of exposure to the intervention, stratified by country. We then used logistic and multiple logistic regression to identify the factors significantly associated with 2 HIV-related behaviors: condom use at last sex and HIV status disclosure. In multivariate analysis, the selection of independent variables was initially determined through literature and theoretical concepts, including socio-demographic characteristics, population types, stigma and discrimination, CD4 testing, relevant risk factors, and exposure to the intervention. Next, only variables that were significant at *p* ≤ 0.15 in bivariate analysis were included in the final multivariate regression analysis. Final model fit was determined using the Hosmer–Lemeshow goodness of fit test. Unadjusted and adjusted odds ratios and 95 % confidence intervals were reported.

#### Effects of PASMO’s Combination Prevention Program

To ascertain effects of the program, we used statistical matching as a quasi-experimental method [21, 22]. We applied coarsened exact matching (CEM) to create statistically equivalent groups among treated cases (exposed to program) and control cases (non-exposure to program) from this observational data. CEM assigns each case into one of a specified set of strata in which members are exactly matched on a set of variables that influence probability of exposure to the program [22–24]. For exposure to mass media, the sample was matched on TV ownership, country, education, and socio-economic status. For exposure to IPC, the sample was matched on sexual identity, country, marital status, and accessing ARV. The selection of matched variables was carefully consulted with program staff. Four key outcomes targeted by the intervention program were included in this effectiveness assessment: condom use at last sex, HIV-status disclosure, adherence to ART, and participation in a support group. Multiple logistic regression controlling for potential covariates were performed using the CEM matched sample.

### Ethical Considerations

The survey was reviewed and approved by the PSI Research Ethics Board (REB). In addition, ethical approvals were obtained from the local IRBs in 3 countries and through consultation with the Ministry of Health in the other 3 countries where the survey was conducted. Interviewers were trained and sensitized on research ethics and data collection, taking into account the sensitive nature of the target population. Written informed consent was obtained for all participants.

## Results

### Description of Socio-demographic Characteristics

Table 1 presents the characteristics of the study population. Participants had a median age of 35 years and relatively low education, with 40 % completing only primary education or less. 44.7 % had some secondary education, and just 12.3 % had attained tertiary education. Participants were also relatively poor, with one-third reporting a monthly income of less than $USD 200 (33.5 %), nearly half reporting earnings of $201–500 (47 %), and only 19.5 % earning more than $500 a month. When using the socioeconomic status (SES) classification applied to Central America (AMAI SEL), a large majority were determined to be in the low (74.7 %), 18.4 % were in the medium, and 6.9 % were in the high SES level. Nearly half of participants were married (44.7 %), 43 % were single or never married, and 12.3 % were separated, divorced, or widowed. One-third of the study population was female (35.8 %), one quarter was males who self-identified as homosexual or bisexual (23.4 %), and 40.8 % of respondents were males who self-identified as heterosexual. About two-thirds of the study participants reported having at least one child. Inter-country differences were small, with Panama and Belize faring better economically.

**Table 1:** Descriptions of the study population (N = 2,818)

Characteristics	Guatemala % (n)	El Salvador % (n)	Nicaragua % (n)	Costa Rica % (n)	Panama % (n)	Belize % (n)	Total
Sample size	903	751	230	248	453	233	2,818
Current age (median)	35.00	35.00	32.00	39.00	34.00	28.00	35.00
Age at HIV diagnosis [median; range]	30 [12–63]	29 [12–70]	28 [14–51]	29 [15–69]	29 [13–71]	21 [1–46]	29 [1–71]
Age							
18–24	8.5 (77)	10.9 (82)	18.7 (43)	12.5 (31)	13.5 (61)	38.6 (90)	13.6 (384)
25–34	36.5 (330)	34.5 (259)	44.4 (102)	25.0 (62)	36.9 (167)	30.9 (72)	35.2 (992)
35–44	31.7 (286)	32.5 (244)	23.9 (55)	28.2 (70)	32.9 (149)	23.2 (54)	30.5 (858)
45+	23.3 (210)	22.1 (166)	13.0 (30)	34.3 (85)	16.7 (76)	7.3 (17)	20.7 (584)
Gender identity							
Female	31.6 (285)	45.7 (343)	31.3 (72)	31.9 (79)	28.0 (127)	44.6 (104)	35.8 (1,010)
Straight males	54.7 (494)	38.3 (288)	39.6 (91)	11.3 (28)	40.6 (184)	27.5 (64)	40.8 (1,149)
Homosexual/bisexual	13.7 (124)	16.0 (120)	29.1 (67)	56.8 (141)	31.4 (142)	27.9 (65)	23.4 (659)
Education							
Primary or less	61.2 (553)	49.4 (317)	23.5 (54)	33.5 (83)	6.0 (27)	21.0 (49)	40.3 (1,137)
Secondary	31.7 (287)	40.2 (302)	67.4 (155)	49.6 (123)	62.7 (284)	67.0 (156)	44.5 (1,256)
Tertiary	7.0 (63)	10.4 (78)	9.1 (21)	16.9 (42)	31.3 (142)	12.0 (28)	12.2 (346)
Socio-economic status							
Low	79.8 (709)	88.6 (661)	85.7 (197)	57.3 (142)	53.9 (242)	57.7 (131)	74.7 (2,082)
Medium	15.1 (134)	9.1 (68)	13.5 (31)	27.4 (68)	30.3 (136)	33.9 (77)	18.4 (514)
High	5.1 (45)	2.3 (17)	0.9 (2)	15.3 (38)	15.8 (71)	8.4 (19)	6.9 (192)
Personal income							
Less than $200	36.8 (294)	50.5 (379)	31.3 (72)	26.0 (61)	14.9 (67)	13.3 (31)	33.5 (904)
$201 to $500	47.2 (377)	43.9 (330)	63.9 (147)	40.4 (95)	44.1 (198)	51.5 (120)	47.0 (1,267)
More than $500	15.9 (127)	5.6 (42)	4.8 (11)	33.6 (79)	41.0 (184)	35.2 (82)	19.5 (525)
Marital status							
Single/never married	31.8 (287)	45.3 (340)	56.1 (129)	59.0 (144)	49.3 (223)	37.0 (85)	43.0 (1,208)
Married/cohabiting	59.4 (536)	50.5 (379)	39.6 (91)	15.6 (38)	29.6 (134)	33.9 (78)	44.7 (1,256)
Separated/divorced/widow	8.9 (80)	4.3 (10)	4.3 (10)	25.4 (62)	21.0 (95)	29.1 (67)	12.3 (346)
Number of children							
None	26.0 (235)	30.4 (228)	48.7 (112)	61.7 (153)	54.5 (247)	48.1 (112)	38.6 (1,087)
1	18.6 (168)	20.1 (151)	14.4 (33)	8.9 (22)	17.7 (80)	10.3 (24)	17.0 (478)
2	21.8 (197)	21.0 (158)	19.1 (44)	12.9 (32)	12.1 (55)	20.2 (47)	18.9 (533)
3 and more	33.6 (303)	28.5 (214)	17.8 (41)	16.5 (41)	15.7 (71)	21.5 (50)	25.6 (720)

### Description of HIV-Related Transmission Risks

Table 2 presents HIV-related transmission risks. A majority of the participants reported using a condom during their last sexual act (86.6 %). More than half reported disclosing their HIV status to their most recent sex partner (55.6 %), and 19.8 % reported having 2 or more sex partners in the past month. A majority of respondents reported having a CD4 test (83.2 %) and a viral load test (74.2 %) in the past 12 months, and over half were on ART treatment (55.1 %). Of those who were taking ART, 86.1 % reported adhering to the daily drug regime. Nearly a quarter reported having STI symptoms in the past 12 months (22.6 %). A small proportion reported being discriminated against by a health care provider in the last 12 months (5.5 %) [*data not shown*], but a much higher proportion reported experiencing any type of abuse (sexual, physical, verbal) (28.1 %), and 12.4 % reported experiencing sexual or physical abuse. Over half of participants reported having been exposed to a PASMO mass media campaign (52.4 %), and a lower proportion reported having received PASMO IPC in a face-to-face session (20.5 %) or via Internet platform (9.5 %) [*data not shown*].

**Table 2:** Description of HIV-related risk factors (N = 2,818)

Characteristics	Guatemala % (n)	El Salvador % (n)	Nicaragua % (n)	Costa Rica % (n)	Panama % (n)	Belize % (n)	Total
Sample size	903	751	230	248	453	233	2,818
Protected sex at last sex	86.6 (780)	87.6 (637)	92.2 (212)	90.6 (213)	89.4 (369)	38.4 (43)	86.1 (2,254)
Women	76.4 (217)	85.3 (278)	91.7 (66)	88.6 (70)	85.6 (95)	25.5 (12)	80.3 (738)
Men	91.2 (563)	89.5 (359)	92.4 (146)	91.7 (143)	90.7 (274)	47.7 (31)	89.2 (1,516)
*Heterosexual*	*91.3 (451)*	*90.5 (256)*	*89.0 (81)*	*88.5 (23)*	*89.2 (157)*	*35.7 (15)*	*88.4 (983)*
*Bisexual/homosexual*	*91.1 (112)*	*87.3 (103)*	*97.0 (65)*	*92.3 (120)*	*92.9 (117)*	*69.6 (16)*	*90.8 (533)*
HIV status disclosure (last sex act)	65.6 (548)	59.7 (360)	46.2 (84)	30.9 (58)	53.0 (123)	28.9 (42)	55.6 (1,214)
Women	79.8 (202)	75.6 (189)	44.9 (22)	41.5 (17)	74.7 (56)	28.9 (15)	69.6 (501)
Men	59.4 (346)	48.4 (171)	46.6 (62)	27.9 (41)	42.7 (67)	28.9 (26)	48.7 (713)
*Heterosexual*	*66.0 (306)*	*58.2 (146)*	*51.3 (40)*	*44.0 (11)*	*49.3 (35)*	*32.1 (17)*	*58.9 (555)*
*Bisexual/homosexual*	*33.6 (40)*	*24.5 (25)*	*40.0 (22)*	*24.6 (30)*	*37.2 (32)*	*24.3 (9)*	*30.3 (158)*
Had multiple sex partners (last 30 days)	12.0 (108)	13.9 (104)	30.4 (70)	36.3 (90)	15.5 (70)	49.4 (115)	19.8 (557)
Women	3.5 (10)	6.1 (21)	27.8 (20)	17.7 (14)	5.5 (7)	44.2 (46)	11.7 (118)
Men	15.9 (98)	20.3 (83)	31.7 (50)	45.0 (76)	19.3 (63)	53.5 (69)	24.3 (439)
*Heterosexual*	*11.3 (56)*	*13.5 (39)*	*17.6 (16)*	*35.7 (10)*	*11.4 (21)*	*56.3 (36)*	*15.5 (178)*
*Bisexual/homosexual*	*33.9 (42)*	*36.7 (44)*	*50.8 (34)*	*46.8 (66)*	*29.6 (42)*	*50.8 (33)*	*39.6 (261)*
Being abused (sexually, physically, verbally) in last 12 months	23.9 (216)	22.6 (170)	21.7 (50)	44.0 (109)	9.7 (44)	87.1 (203)	28.1 (792)
Women	30.5 (87)	23.9 (82)	20.8 (15)	51.9 (41)	11.0 (14)	86.5 (90)	32.6 (329)
Men	20.9 (129)	21.6 (88)	22.2 (35)	40.2 (68)	9.2 (30)	87.6 (113)	25.6 (463)
*Heterosexual*	*17.6 (87)*	*16.0 (46)*	*17.6 (16)*	*35.7 (10)*	*10.9 (20)*	*90.6 (58)*	*20.6 (237)*
*Bisexual/homosexual*	*33.9 (42)*	*35.0 (42)*	*28.4 (19)*	*41.1 (58)*	*7.0 (10)*	*84.6 (55)*	*34.3 (226)*
Had a CD4 test (last 12 months)	88.5 (799)	90.0 (676)	82.2 (189)	91.1 (226)	76.6 (347)	46.4 (108)	83.2 (2,345)
Had a viral load test (last 12 months)	77.6 (701)	77.0 (578)	79.6 (183)	89.9 (223)	71.5 (324)	35.2 (82	74.2 (2,091)
Under ART treatment	81.5 (715)	86.2 (638)	67.6 (152)	91.1 (224)	75.8 (313)	41.6 (69)	79.2 (2,111)
Adherence to ART	92.9 (663)	95.1 (607)	89.5 (136)	78.1 (175)	68.3 (205)	23.8 (15)	86.1 (1,801)
Attended an HIV support group	20.6 (186)	40.9 (307)	14.8 (34)	60.1 (149)	11.7 (53)	25.8 (60)	28.0 (789)

Among men, homosexual men and straight men show different HIV-related risk and therefore, they were further being analyzed and presented separately (in italic)

When stratifying the data by gender (male and female), and sexual orientation (within the male category), men were more likely than women to use a condom or to have multiple sex partners, while men were less likely to disclose HIV status to their sex partner or to experience physical and mental violence. Further, homosexual and bisexual men were more likely to experience violence or to have multiple sex partners compared to heterosexual men. (31 transgender women in the sample were collapsed in the homosexual men category).

When examining data across the 6 countries, study participants who lived in Belize reported significantly higher levels of sexual risk behaviors and lower levels of health seeking behaviors than those who lived in other 5 countries. Respondents living in Belize were less likely to report: using a condom, disclosing their HIV-positive status to their last sexual partner, having had a CD4 or viral load test, and being on ART. They were more likely to report: multiple sex partners, STI symptoms, and experiencing abuse. In addition, PLHIV in Belize reported a significant higher prevalence of any type of violence compared to other 5 countries in the region.

### Factors Associated with Condom Use at Last Sex (Bivariate and Multivariate Analysis)

Table 3 presents factors associated with condom use at last sex. Bivariate analysis findings suggest that age, gender identity, education, SES, HIV disclosure, violence, currently being on ART, and having had a CD4 test are associated with condom use. In multivariate analysis, after controlling for country and SES, we found that heterosexual men (AOR = 1.9; 95 % CI 1.3–2.7) and homosexual/bisexual men (AOR 2.5:1.6–4.0) had higher odds of condom use compared to women. Participants who disclosed their HIV status to their last sex partner were more likely to use a condom (AOR = 1.8:1.3–2.5). Notably, those who were currently receiving ART (AOR = 2.1:1.5–3.0) and had a CD4 test at least once in the past 12 months (AOR = 1.16:1.03–2.5) were more likely to use condoms. Victims of physical or sexual violence (AOR = 0.25:0.2–0.33), and those who had STI symptoms in the last 12 months (AOR = 0.43:0.33–0.55) were less likely to use condoms.

**Table 3:** Factors associated with condom use at last sex

Variables	Condom use at last sex	
	Odds ratio (95 % CI)	Adjusted odds ratio (95 % CI)
Age		
18–34 years old	1.0	1.0
≥35 years old	5.1 (4.4–5.9)***	1.0 (0.7–1.4)
Gender identity		
Females	1.0	1.0
Heterosexual males	1.9 (1.5–2.4)***	1.9 (1.3–2.7)***
Homosexual/bisexual males transgender	2.4 (1.8–3.3)***	2.5 (1.6–4.0)***
Education		
Primary or less	1.0	1.0
Secondary	1.2 (0.9–1.5)	1.4 (1.0–1.9)
Tertiary	2.5 (1.6–3.9)***	1.4 (0.7–2.9)
Medium or high SES	1.4 (1.1–1.9)*	1.3 (0.8–2.0)
Marital status		
Single	1.0	N/A
Married/cohabiting	1.1 (0.9–1.4)	
Separated/divorced/widow	0.7 (0.5–1.0)	
Had multiple sex partners in last 30 days	0.9 (0.7–1.2)	N/A
Disclosed HIV status to the last sex partner	1.7 (1.3–2.2)***	1.8 (1.3–2.5)***
Suffered physical or sexual abuse in last 12 months	0.25 (0.2–0.33)***	0.5 (0.3–0.8)**
Under ART treatment	2.4 (1.8–3.1)***	2.1 (1.5–3.0)***
Had a CD4 test in last 12 months	2.7 (2.0–3.6)***	1.6 (1.03–2.5)*
Pseudo R2		13 %

Country was controlled for in all of the multivariate regression models

Multivariate analysis for the variable condom use at last sex was performed on an analytical sample of 1,954 (respondents with missing value were dropped from the model)

* *p* < 0.05, ** *p* < 0.01, *** *p* < 0.001

### Factors Associated with HIV-Status Disclosure (Bivariate and Multivariate Analysis)

Table 4 presents factors associated with HIV-status disclosure. Bivariate analysis findings suggest gender identity, education, SES, marital status, having multiple sex partners, and being physically or sexually abused are significant correlates. In multivariate analysis controlling for SES and country, married participants (AOR = 5.3:4.3–6.7) were more likely to disclose HIV status. As compared to women, heterosexual men (AOR = 0.6:04–0.7) and homosexual men (AOR = 0.4:03–0.6) were less likely to disclose HIV status. Those reporting multiple sex partners (AOR = 0.3:0.1–0.4) were also less likely to disclose HIV status to their last sex partner.

**Table 4:** Factors associated with HIV-status disclosure

Variables	HIV disclosure (N = 2,076)	
	Odds ratio (95 % CI)	Adjusted odds ratio (95 % CI)
Age		
18–34 years old	1.0	N/A
≥35 years old	1.0 (0.8–1.2)	
Gender identity		
Females	1.0	1.0
Heterosexual males	0.6 (0.5–0.8)***	0.6 (0.4–0.7)***
Homosexual/bisexual males transgender	0.2 (0.15–0.24)***	0.4 (0.3–0.6)***
Education		
Primary or less	1.0	1.0
Secondary	0.7 (0.6–0.8)***	1.2 (1.0–1.6)
Tertiary	0.5 (0.4–0.7)***	1.0 (0.7–1.6)
Medium or high SES	0.8 (0.6–0.9)**	1.3 (1.0–1.8)
Marital status		
Single	1.0	1.0
Married/cohabiting	6.6 (5.4–8.1)***	5.3 (4.3–6.7)***
Separated/divorced/widow	1.4 (1.1–1.9)*	1.2 (0.8–1.7)
Used condom at last sex	1.7 (1.3–2.2)***	1.8 (1.3–2.5)***
Multiple sex partners in the last 30 days	0.17 (0.14–0.21)***	0.3 (0.2–0.4)***
Suffered physical or sexual abuse in the last 12 months	0.5 (0.4–0.6)***	0.7 (0.5–1.01)
Under ART treatment	1.0 (0.8–1.2)	0.8 (0.6–1.04)
Pseudo R2		22 %

Country was controlled for in all of the multivariate regression models

Multivariate analysis was performed on an analytical sample of 2,076 (respondents with missing value were dropped from the regression model)

* *p* < 0.05, ** *p* < 0.01, *** *p* < 0.001

### Effects of PASMO’s Behavior Change Communications Intervention (CEM Analysis)

Results from the multivariate regression analysis of the CEM matched sample are presented in Table 5. We found exposure to PASMO’s mass media intervention was associated with increased condom use (AOR = 1.8; 95 % CI 1.3–2.5), participation in self-help groups (AOR = 1.4; 95 % CI 1.2–1.8), and HIV-status disclosure (AOR = 1.5; 95 % CI 1.2–1.9). IPC interventions also significantly increased condom use (AOR = 2.7; 95 % CI 1.7–4.3) and participation in support groups (AOR = 4.4; 95 % CI 3.5–5.6). We did not find a statistically significant impact of the IPC intervention on ART adherence.

**Table 5:** Program effects: logistic regression using CEM matched samples

Outcomes	Exposed to mass media (N = 2,134)	Exposed to interpersonal communications (IPC) (N = 2,299)
	Adjusted odds ratio (95 % CI)	Adjusted odds ratio (95 % CI)
Condom use at last sex	1.8 (1.3–2.5)***	2.7 (1.7–4.3)***
HIV status disclosure	1.5 (1.2–1.9)***	1.2 (1.0–1.5)
Participation in a self-help group	1.4 (1.2–1.8)***	4.4 (3.5–5.6)***
ART adherence	1.0 (0.7–1.4)	1.4 (1.0–2.1)

For mass media, the sample was matched on: TV ownership, country, education, and socio-economic status

For IPC, the sample was matched on: gender identity, country, marital status, and accessing ARV

Effect of the intervention on each outcome was assessed using multiple logistic regression analysis, adjusting for SES variable (if not matched). In addition, “ART treatment” and “HIV disclosure” were also adjusted for in the “condom use” model, and “violence” and “number of sex partners” were also adjusted for in the “HIV-disclosure” model

* *p* < 0.05, ** *p* < 0.01, *** *p* < 0.001

## Discussion

This first regional-level study describes PLHIV in Central American and factors associated with key HIV-related behaviors, i.e. condom use and HIV-status disclosure. It also provides insights into the mid-term effectiveness of behavioral interventions on factors associated with on-going HIV transmission risk.

### HIV-Related Transmission Risks

Findings suggest significant gender disparities with regards to HIV-transmission risks and being a victim of violence. In particular, women were significantly less likely to use condoms, while they were more likely to report being abused. However, men reported having more sex partners and lower HIV-status disclosure to sex partners. Experiences of violence were common, particularly among women and homosexual men. Studies in Central America and around the world suggest that women and homosexual men suffer substantial levels of violence [25–28]. Regardless of on-going interventions targeting health care providers who provide services for PLHIV and stigmatized populations, a small proportion of PLHIV still reported being discriminated against by a health care provider. In the context of the PHDP framework, policies and programs should continue to ensure equal access to non-discriminatory health services.

### Factors Associated with Condom Use

We found a set of factors that influence condom use, including gender, HIV-status disclosure, violence, ART, and having a regular CD4 count test. Both heterosexual and homosexual men were more likely than women to use condoms. Previous research has shown that women often have difficulty negotiating condom use or asking for a condom due to power imbalances, fear of violence, and gender inequality [29, 30], therefore increasing their susceptibility for HIV and STIs. After controlling for key covariates, sexual and physical violence were found to be barriers to condom use. This finding is aligned with previous research that shows a negative impact of violence on sexual risk behavior [31, 32]. Violence limits a woman’s ability to negotiate condom use and also has negative consequences on mental and physical health that could ultimately complicate the victim’s health behaviors [29]. Studies from many countries have found that violence is associated with increased HIV and STI prevalence [32–35]. The high rates of violence and crime in Central America have been well documented, including high levels of gender-based violence, and complacency around violence [36]. Interventions to raise awareness about this issue and to reduce violent acts are critical for the successful implementation of any HIV intervention in Central America.

The study also supports the importance of health seeking behaviors in reducing HIV risk among PLHIV. Currently on ART, participation in a self-help group, and regular monitoring of CD4 increased condom use. This is consistent with findings from a number of other studies assessing the impact of ART on sexual risk behaviors among PLHIV in resource-poor settings [15, 37, 38]. We offer a few explanations for this. First, individuals on ART, monitoring CD4 counts, or participating in support groups likely receive risk-reduction counseling from their health care providers or peer outreach workers. Second, being part of the care system likely helps PLHIV receive emotional support and improved physical and mental health outcomes that might foster positive behaviors [37, 38]. Third, ART may have an impact on a set of indicators: HIV disclosure was found to be associated with condom use, while both condom use and HIV disclosure were positively influenced by ART (discussed more in the next heading). These findings have critical implications for policies, as the combined effects of both reducing HIV viral loads and HIV transmission risks would yield synergistic impacts on reducing HIV transmission.

### Factors Associated with HIV-Status Disclosure

Consistent with other studies, we found gender differences in HIV disclosure [15, 39]. Both heterosexual and homosexual men were less likely than women to disclose their HIV status to their sex partners. This may suggest women are less likely than men to engage in extramarital sex or sex with multiple partners, and thus the relationship with their male partner is solid enough for disclosure. This might also be because women are less likely to ask male partners their HIV status due to having less power or because men do not feel obliged to disclose [15].

In addition, our analysis suggests that a set of risk behaviors might be nested within one another. In particular, we found PLHIV with multiple sex partners were less likely to disclose HIV status; and those who did not disclose were less likely to use condoms. We hypothesized that PLHIV might not feel responsible for protecting their partners in a casual relationship and thus choose not to disclose their HIV status, or they may fear being discriminated against or fear that that their sexual encounter might be interrupted. Low disclosure among heterosexual and homosexual men, together with having a larger number of sex partners, would intensify HIV transmission among MSM or put women at higher risk of being infected. These findings imply that knowing one’s partner’s HIV status is vital and sero-sorting might be an effective HIV prevention strategy.

### Effects of PASMO’s Combination Prevention Program

The findings indicate that 2 years into the implementation, PASMO’s program has reached a moderate proportion of PLHIV in 6 countries in Central America. Condom use was high, and most PLHIV surveyed were on ART treatment and having their CD4 monitored regularly. Among those who were on ART, most reported that they were able to adhere to the treatment regime. About one-third of the sample reported participating in a self-help group.

The program has made positive effects on a number of targeted outcomes. When stratifying the analysis by intervention channels, mass media significantly increased the odds of condom use by 1.8 times, HIV disclosure by 1.5 times, and participation in a self-help group by 1.4 times. IPC has made positive impacts on condom use and participation in self-help groups by an even larger magnitude compared to mass media intervention messaging. We did not find effects of either mass media or IPC on ART adherence; and IPC also had no effect on HIV disclosure. This is probably because the messaging around ART adherence was not easily communicated by non-health communications channels, or perhaps it may take more time to have an effect. We also found no effect of both mass media and IPC on number of sex partners [*data not shown*].

The findings suggest that behavioral interventions in the larger context of a combination prevention program have positive effects on reducing HIV transmission risk among PLHIV. This approach ensures that intervention programs targeting PLHIV address their comprehensive needs, including access to care and treatment, legal support, social and emotional support, and counseling on gender-based violence. We hypothesize that the synergistic overlap of multi-component programming, like PASMO’s Combination Prevention Program might have contributed to the impact. We acknowledged the limitation of this data, which only allow us to examine the effects of the behavioral interventions. Exposure to the biomedical and structural components, in particular referrals to legal services, stigma reduction, and STI treatment, were either low or not captured comprehensively in this mid-term survey data. We expect that our end-line data will capture the exposure of the biomedical and structural interventions and help test this hypothesis.

There is minimal published data on the effectiveness of behavioral interventions among PLHIV [40–42]. These limited data, however, suggest that our findings are in line with other studies. Several meta-analyses and systematic reviews show that targeted behavior change communications are effective in reducing unsafe sex and increasing HIV status disclosure among PLHIV [40–42]. In addition, the effect size of interventions among PLHIV is even larger than that of interventions targeting HIV-negative populations. However, most of these studies are from the US or developed countries, or part of the HIV testing and counseling programs [40, 42]. A research gap remains in regard to effectiveness evidence of behavioral interventions for PLHIV, especially under the combination prevention framework. This research gap might be the result of the lack of behavioral interventions and the particularly high prioritization of ART among PLHIV.

## Limitations

The study has a number of limitations. First, we recruited participants from clinics and support groups, and therefore participants are likely not representative of all PLHIV in Central America. This may mean we missed PLHIV who were not yet linked to care and treatment. Furthermore, PLHIV attending clinics would likely have to go through counseling for risk reduction and ART adherence and thus their risk behaviors would likely be lower and adherence higher than the general population of PLHIV. This limitation, however, is justified, as recruiting a representative sample of PLHIV is problematic due to the small population size and non-existence of a sampling frame. Attempts to get a representative sample of PLHIV would pose ethical challenges around the protection of confidentiality and privacy, particularly important for PLHIV.

Second, the data are susceptible to a number of biases such as social desirability, recall, and non-response biases. Participants might over-report the use of condoms, HIV status disclosure, and ART adherence, while under-reporting number of sex partners, thus impacting the analytical power. Response is also likely to be subjected to recall bias, particularly around exposure to intervention activities. There is, however, little evidence that there is a difference in reporting of sexual behavior between those exposed and not exposed to the interventions. In addition, our analysis showed that non-response in this study was minimal (2 % and less for all key variables) in both exposed and non-exposed groups.

Third, the study did not use an experimental design and thus findings on intervention effectiveness should be interpreted with caution. However, the large sample size and the use of CEM could potentially improve the confidence in our findings. This approach is particularly important, as it utilizes a single cross-sectional data while reflecting the real world context of public health interventions where evaluation should not be seen as a factor that slows down or intervenes with the program implementation.

## Conclusions

PLHIV in Central America are economically disadvantaged compared to the general population, and their HIV risks and vulnerability vary significantly by gender, sexual orientation, marital status, age, and country. Targeted interventions to reduce secondary HIV transmission among PLHIV remain critical and should be tailored accordingly. In particular, raising awareness of and reducing violence towards women and MSM are critical; while reducing number of sex partners and increasing HIV disclosure are important for men and those who are young or single. Among the 6 countries where PASMO’s Combination Prevention Program is implemented, a greater emphasis should be placed on Belize, where intervention coverage and access to ART and CD4 tests is low, and violence and risky behaviors are extremely high. In addition, gender-based violence should be examined under the lens of gender identities, and more research on this topic is needed to gain a better understanding of how the perceptions of masculinity and widespread homophobia might play a role, particularly among women and MSM living with HIV [43, 44]. Besides the proven impact of ART on lowering transmission probability, it can also lower HIV-related risk behaviors. Finally, behavioral interventions in the larger context of a comprehensive prevention intervention for PLHIV are effective and should be continued to be promoted.

## Electronic supplementary material

Below is the link to the electronic supplementary material.

Supplementary material 1 (DOCX 11 kb)
