# Supplementary material for: Effectiveness of Behavior Change Communications for Reducing Transmission Risks Among People Living with HIV in 6 Countries in Central America
Source: AIDS Behav. 2014 Oct 5;19(7):1203–13. doi: 10.1007/s10461-014-0910-0 (PMC4503879; doi:10.1007/s10461-014-0910-0)
Supplement: Supplementary file 1 — Supplementary material 1 (DOCX 11 kb) [file 10461_2014_910_MOESM1_ESM.docx]

**Supplemental Table 1: Details of survey locations and sample size**

| **Country** | **City** | **n** | **%** |
| --- | --- | --- | --- |
| Guatemala | Coatepeque | 277 | 30.7 |
|  | Guatemala City | 451 | 49.9 |
|  | Puerto Barrios | 175 | 19.4 |
|  | ***Total*** | ***903*** | ***100.0*** |
| El Salvador | Santa Ana | 92 | 12.3 |
|  | Cuscatlán | 38 | 5.1 |
|  | San Salvador | 503 | 67.0 |
|  | Usulután | 49 | 6.5 |
|  | San Miguel | 69 | 5.2 |
|  | ***Total*** | ***751*** | ***100.0*** |
| Nicaragua | ***Managua*** | ***230*** | ***100.0*** |
| Costa Rica | San José | 150 | 60.5 |
|  | Alajuela | 16 | 6.5 |
|  | Heredia | 9 | 3.6 |
|  | Cartago | 56 | 22.6 |
|  | Puntarenas | 17 | 6.9 |
|  | ***Total*** | ***248*** | ***100.0*** |
| Panamá | ***Panamá City*** | ***453*** | ***100.0*** |
| Belize | Belize City | 49 | 21.0 |
|  | Orange Walk | 47 | 20.2 |
|  | Cayo | 92 | 39.5 |
|  | Stann Creek | 45 | 19.3 |
|  | ***Total*** | ***233*** | ***100.0*** |
